# Postvitrectomy endophthalmitis caused by *Morganella morganii*: a case report and literature review

**DOI:** 10.1186/s12879-022-07248-y

**Published:** 2022-03-18

**Authors:** Chung-Ting Wang, Yin-Hsi Chang, Kuan-Jen Chen, Hung-Da Chou

**Affiliations:** 1 Department of Medical Education, Chang Gung Memorial Hospital, Linkou Medical Center, Taoyuan City, Taiwan; 2 Department of Ophthalmology, Chang Gung Memorial Hospital, Linkou Medical Center, No. 5, Fuxing St., Gueishan Dist., Taoyuan City, 333 Taiwan; 3 College of Medicine, Chang Gung University, Taoyuan City, Taiwan

**Keywords:** Postoperative endophthalmitis, *Morganella morganii*, Retrolental exudative membrane, Vitrectomy, Silicone oil

## Abstract

**Background:**

Postvitrectomy endophthalmitis is a rare and serious complication following vitreoretinal surgeries. *Morganella morganii*, an emerging gram-negative, facultative anaerobic rod, is related to severe nosocomial infections in various organs and thus has gained importance in recent decades. *Morganella morganii* infection following intraocular surgery is rarely reported.

**Case presentation:**

We report an immunocompetent patient with *Morganella morganii*-related endophthalmitis after vitrectomy for retinal detachment who presented with hand motion visual acuity, hypopyon and a unique retrolental exudative membrane. Initially, the patient was unresponsive to empirical intravitreal ceftazidime and vancomycin but recovered with vision preservation (20/63) after surgical removal of the membrane and silicone oil tamponade.

**Conclusions:**

*Morganella morganii* intraocular infection is often devastating, likely due to its high multidrug-resistance rate via intrinsic ß-lactamase production, multiple acquired traits related to additional genetic mechanisms, and fimbrial adhesion, urease production, and type III secretion system-associated biofilm formation. The above characteristics of *M. morganii* may lead to an inadequate response to empirical intravitreal antibiotics, and early surgical intervention should be considered.

## Background

Postoperative endophthalmitis is a rare complication, with an estimated incidence of 0.05% [1]. Following the development of microincision vitrectomy surgery, the average incidence of postoperative endophthalmitis was reported to be 0.03–0.11% [1]. Most of the causative pathogens of postoperative endophthalmitis are gram-positive bacteria (45.9–97%), including *Staphylococcus aureus* and coagulase-negative staphylococci [2, 3]. As a gram-negative facultative anaerobic rod, *Morganella morganii* is associated with multi-drug resistance and high morbidity and mortality, especially in immunocompromised patients and neonates [4, 5]. However, *M. morganii* has rarely been reported as a cause of infectious ocular disease. Visual outcomes associated with this pathogen have generally been poor [6–10].

Here, we report a case of postvitrectomy endophthalmitis caused by *M. morganii*, which had a unique presentation of a retrolental exudative membrane. Early vitrectomy with membrane removal and silicone oil tamponade controlled the infection and preserved the vision of the patient. The purpose of this case report is to highlight the role of early surgical intervention in such conditions related to rare pathogens.

## Case presentation

A 48-year-old man with chronic hepatitis B had a history of self-limited idiopathic intermediate uveitis in the left eye and high myopia in both eyes. He visited our clinic due to rhegmatogenous retinal detachment in the left eye and underwent segmental scleral buckling. His vision recovered to 20/25 after the operation, with good reattachment of the retina.

Six months later, he presented to our clinic with progressive visual field defects in the left eye. Best-corrected visual acuity was 20/25 in the left eye, and observations under slit-lamp biomicroscopy were unremarkable. Dilated fundus examination revealed a redetachment of the retina and a reopening of the previous retinal break. Recurrent rhegmatogenous retinal detachment was diagnosed, and the patient underwent standard three-port 23-gauge pars plana vitrectomy to reattach the retina. During the operation, triamcinolone acetonide (Tai Yu Chemical & Pharmaceutical Co., Hsinchu, Taiwan) was used to assist in the visualization of the vitreous and was washed out during the surgery. The operation went smoothly, and 10% C_3_F_8_ was injected for vitreous tamponade. Postoperatively, the patient was asked to maintain prone positioning for 2 weeks and was prescribed 1% prednisolone eyedrops (Alcon Research., Texas, USA) four times per day, along with 1% atropine and dexamethasone + tobramycin ointment (Tobradex, Alcon, Puurs, Belgium) twice per day.

**Fig. 1:**
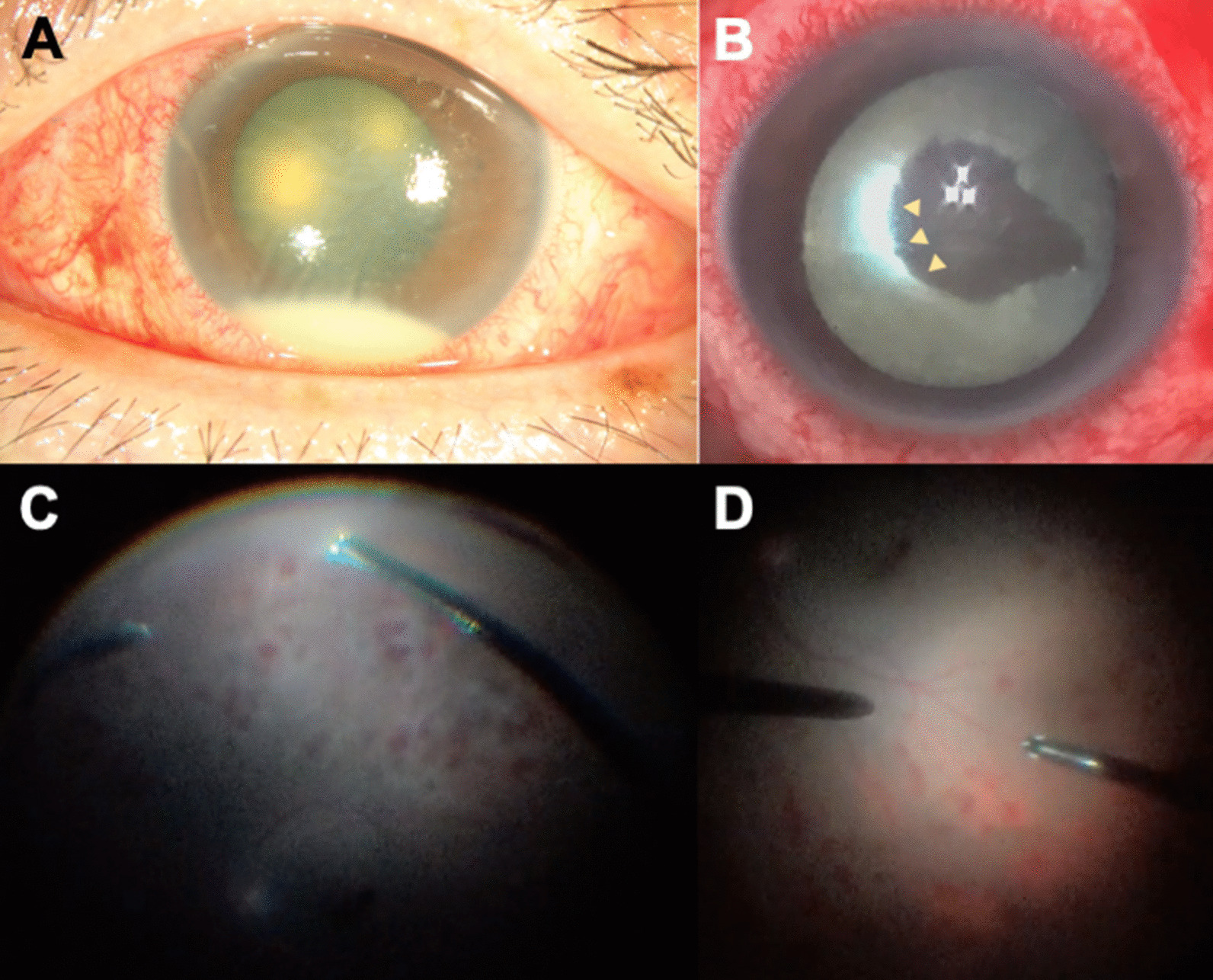
**A** External eye photograph shows an inflamed eye with hypopyon in the anterior chamber. **B** During rescue vitrectomy, a dense retrolental membrane was identified. Arrowheads mark the margin of the remaining membrane during removal. **C** The fundus could be examined after the removal of the exudative membrane, which showed diffuse retinitis. **D** The macula was mostly spared, likely due to gas tamponade and prone positioning

On postoperative day 2, whitish deposits and membrane-like exudate over the posterior lens surface were noted. The patient had no discomfort. Hourly 0.5% levofloxacin eyedrops (Cravit, Santen Pharmaceutical Co., Osaka, Japan) were prescribed. On postoperative day 3, the patient began to complain of tenderness over the periorbital area, and visual acuity dropped to hand movement. The patient’s intraocular pressure was 14 mmHg. Slit-lamp examination showed congested conjunctiva, corneal edema and 1 + cell and flare in the anterior chamber with a 2 mm hypopyon (Fig. 1A). The fundus was obscured. Ocular ultrasonography showed intravitreal gas without obvious vitreous opacity. Postoperative endophthalmitis was suspected based on the clinical presentation. Intravitreal empirical antibiotics with ceftazidime 2 mg/0.05 ml (China Chemical & Pharmaceutical Co., Tainan, Taiwan) and vancomycin 1 mg/0.05 ml (Sandoz, Gentle Pharma Co., Yunlin, Taiwan) were injected, and vitreous fluid samples were sent for microorganism culture. In addition, the results of laboratory work-ups, including complete blood and biochemistry panels; a lipid profile; urinalysis; glycohemoglobin, human leukocyte antigen-B27, antinuclear antibody, angiotensin converting enzyme, human immunodeficiency virus antigen and antibody level tests; and syphilis tests, were all unremarkable.

On postoperative day 4, there was no clear resolution of the symptoms, and a prominent anterior chamber reaction and vitreous opacity persisted. On postoperative day 5, vitreous fluid culture yielded moderate growth of *Morganella morganii*, leading to a decision to perform vitrectomy on the same day. During surgery, a dense retrolental membrane was noted, and we were able to remove the membrane with a vitrectomy probe without damaging the lens (Fig. 1B). The fundus was then examined in detail, which revealed diffuse patchy intraretinal hemorrhages that spared the macula (Fig. 1C, D). There was no retinal redetachment or retinal necrosis. Intravitreal ceftazidime 1 mg/0.05 ml and triamcinolone 0.1 mg/0.1 ml were administered, and silicone oil was injected as an inert medium to prevent pathogen growth. A subsequent drug sensitivity test showed that the pathogen was resistant to cefazolin and cefuroxime but was sensitive to 3rd- and 4th-generation cephalosporins and ciprofloxacin.

After vitrectomy, the ocular infection gradually subsided over 1 week. Silicone oil was removed 5 months later, and the retina remained attached. Despite the formation of posterior subcapsular cataracts, the patient’s vision recovered to 20/63.

## Discussion and conclusions

Risk factors for the development of postvitrectomy endophthalmitis include immunosuppressed status, diabetes mellitus, preoperative or perioperative use of steroids, nonsilicone oil-filled eyes, and sutureless sclerotomies [1, 3, 11–13]. Ninety-one percent of cases of postvitrectomy endophthalmitis development occurred in the first 5 postoperative days, and the most common sign was hypopyon [11]. Intravitreal antibiotic injection is the first-line treatment for postvitrectomy endophthalmitis, but the adoption of early vitrectomy to improve visual outcomes has been explored in recent studies [2, 14].

The visual outcome of postvitrectomy endophthalmitis is variable but is generally poor in most cases [1, 3, 11, 12]. Gram-negative organism infections are relatively rare following ocular surgery, especially following vitreoretinal surgeries (0–15.3%) [3, 12]. Nevertheless, gram-negative bacteria may cause extensive damage to ocular tissues due to the highly virulent toxins and proteolytic enzymes these organisms produce [2, 11].

*Morganella morganii*, a gram-negative, facultative anaerobic rod of the *Enterobacteriaceae* family, can be found in the natural environment and can colonize the human, mammalian, or reptile gastrointestinal tract. The pathogen was initially found to cause mild summer diarrhea; before the 1990s, there were only scattered case reports of *M. morganii*-related infections. In recent decades, cases have increasingly been reported, and it was demonstrated that the pathogen can further cause severe nosocomial infections, including sepsis and hepatobiliary, urinary tract, and wound infections [4, 5, 15, 16]. In a 6-year study in a tertiary hospital, *M. morganii* ranked as the ninth most common pathogen (1.5%) among all gram-negative nosocomial infections [15]. Furthermore, in a recent study in Taiwan, 46.2% of nosocomial *M. morganii* infections in the intensive care unit were resistant to imipenem [17]. In a multicenter study of 73 medical centers from 37 countries, *M. morganii* showed 100% resistance to ceftazidime but 50% sensitivity to ciprofloxacin and 66.7% sensitivity to levofloxacin isolated from intraabdominal infection [16]. The high rate of drug resistance develops due to the pathogen’s intrinsic inducible AmpC β-lactamase production and multiple acquired traits related to additional genetic mechanisms, such as horizontal gene transfer from other pathogens, ultimately leading to extended-spectrum β-lactamase (ESBL)-producing and other novel resistant strains of *M. morganii* [4, 5, 16]. Due to the high morbidity and mortality and a broad spectrum of pathogenicity in various organs, *M. morganii-*related infection has gained increasing clinical importance [4, 5, 15].

Ocular infection caused by *M. morganii* is rare [4]. In the literature, only ten *M. morganii*-related endophthalmitis cases have been reported in the past 30 years [4, 5, 18, 19]. Cases with details reported in the literature are listed in Table 1. Among these cases, two occurred after cataract surgery [6, 7], one after trabeculectomy [10], and one presented with endogenous endophthalmitis [9]. Only one case developed after vitrectomy for epiretinal membrane peeling, with an unfavorable outcome in hand motion visual acuity under intravitreal antibiotic treatment [8]. The prognosis of *M. morgani*i endophthalmitis is poor, with final visual acuity ranging from no light perception to hand motion [6–10].

The current patient presented with hypopyon along with a dense retrolental exudative membrane two days after vitrectomy. The formation of the retrolental membrane might be explained by inflammatory debris deposition, along with *M. morganii*’s traits of fimbrial adhesion, urease production, and type III secretion system-associated biofilm formation [4, 15], which has been reported in postimplantation osteomyelitis patients [20]. We speculate that gas tamponade with prone positioning after surgery limited the extent of bacterial spread in our patient and likely led to the accumulation of bacteria and the formation of an exudative membrane in the retrolental space.

The timing and effectiveness of vitrectomy for postoperative endophthalmitis according to the presenting visual acuity have long been discussed. With the development of wide-angle viewing systems and microincision surgical instruments, early intervention with improved visual outcomes has been noted in recent studies [2, 14]. Furthermore, biofilm formation by bacteria can lead to segregation of the vitreous cavity and has been proposed as an important reason for unresponsiveness to intravitreal antibiotic injections [2]. If no improvement is observed 48 hours after empirical antibiotic intravitreal injection, prompt vitrectomy is recommended [2]. This was what we observed in our patient, who did not respond well initially to intravitreal ceftazidime and vancomycin until the dense exudative membrane was surgically removed during the subsequent vitrectomy.

However, the isolated strain of *M. morganii* in our case showed susceptibility to 3rd- and 4th-generation cephalosporins and ciprofloxacin, which is not an ESBL strain, making initial empirical intravitreal ceftazidime a reasonable choice. Single intravitreal ceftazidime without vancomycin was injected during vitrectomy based on a preliminary report of *M. morganii* isolation on postoperative day 5. Removal of the membrane, proper selection of antibiotics, silicone oil tamponade and prone positioning are possible reasons for the successful vision preservation for our patient. Silicone oil tamponade after vitrectomy creates a potential hydrophobic bactericidal environment, limiting inflammation by inhibiting inflammatory cell migration and microorganism contact with the intraocular structures [21]. Prone positioning also concentrates antibiotics and protects the macula from drug toxicity [21].

In conclusion, we report the case of an immunocompetent patient with postvitrectomy endophthalmitis who presented with hypopyon and a dense retrolental membrane caused by infection with *M. morganii*, an emerging gram-negative pathogen that rarely causes intraocular infection. Intravitreal antibiotic injection alone might not be sufficient to control *M. morganii*-related endophthalmitis in consideration of the nature of its virulence, high rate of multidrug resistance, and formation of a unique retrolental exudative membrane. Early rescue vitrectomy with removal of the retrolental membrane may have been the key factor for our patient to control infection and ensure vision preservation.

**Table 1:** Details of *Morganella morganii*-related endophthalmitis reported in the past 30 years

	Age/Sex	Comorbidity	Eye	Scenario	Clinical presentation	Visual acuity at onset	Intervention	Final visual acuity
Cunningham et al. (1997) [6]	68/F	Diabetes mellitus	N/A	3 days after uncomplicated cataract surgery	Corneal edema, 4 + AC cells, flare and hypopyon	Hand movement	1. Intravitreal CAZ + VAN and dexamethasone 2. Subconjunctival CAZ + VAN 3. Intravenous CAZ + VAN 4. Topical hyoscine and steroid 5. Vitrectomy for recurrence	20/80 after first episode, down to counting fingers after recurrence
Tsanaktsidis et al. (2003) [7]	84/F	Subclinical urinary tract infection with *Escherichia coli *and multiresistant *Acinetobacter* species	OS	2 days after cataract surgery complicated with posterior capsule tear	Ocular pain, conjunctival injection, corneal edema, 4 + AC cells, flare and hypopyon	Hand movement	1. Intravenous AMK + VAN → shift to timentin 2. Topical and oral CPFX, with topical corticosteroids	No light perception, evisceration
Zaninetti et al. (2003) [8]	65/F	Prior retinal detachment	OD	3 days after vitrectomy for epiretinal membrane peeling	Conjunctiva hyperemia, corneal edema, hypopyon	Hand movement	1. Intravenous OFX + IPM 2. Topical CHL + GEN 3. Intravitreal CAZ + VAN and dexamethasone	Hand movement
Christensen et al. (2004) [9]	80/F	Nil	OU	Endogenous endophthalmitis 1 week after left total hip alloplasty-related sepsis	Conjunctival injection, corneal edema, AC fibrinous exudate, posterior synechiae	Light perception	1. Intravenous CXM → shift to CPFX Vitrectomy 2. Intraviteal injection with CAZ + VAN + GEN + AMB (OD); CAZ + VAN + dexamethasone (OS)	No light perception
Kuang et al. (2008) [10]	74/M	Pulmonary tuberculosis Gastric ulcer Hyperthyroidism	OS	1 day after trabeculectomy for uncontrollable IOP after eyeball rupture	Severe pain, purulent bleb, eyelid edema, diffuse whitish exudates in AC, elevated IOP	3/60	1. Topical CAZ, VAN and corticosteroids 2. Intravenous and subconjunctival AMK + CEZ 3. Intravitreal CAZ + VAN 4. Wound debridement	No light perception
Wang et al. (current case)	48/M	Chronic hepatitis B Idiopathic intermediate uveitis Prior retinal detachment	OS	2 days after vitrectomy for recurrent retinal detachment	Periorbital tenderness, hypopyon, retrolental exudative membrane, peripheral retinal vasculitis	Hand movement	1. Topical LVX 2. Intravitreal CAZ + VAN 3. Vitrectomy with removal of the retrolental biofilm, intravitreal CAZ and triamcinolone	20/63

## Data Availability

The datasets used and/or analysed during the current study are available from the corresponding author on reasonable request.

## Abbreviations

AC: Anterior chamber
ESBL: Extended-spectrum β-lactamase
IOP: Intraocular pressure
AMB: Amphotericin B
AMK: Amikacin
CAZ: Ceftazidime
CEZ: Cefazolin
CHL: Chloramphenicol
CPFX: Ciprofloxacin
CXM: Cefuroxime
GEN: Gentamicin
IPM: Imipenem
LVX: Levofloxacin
OFX: Ofloxacin
VAN: Vancomycin
